# Reachy, a 3D-Printed Human-Like Robotic Arm as a Testbed for Human-Robot Control Strategies

**DOI:** 10.3389/fnbot.2019.00065

**Published:** 2019-08-14

**Authors:** Sébastien Mick, Mattieu Lapeyre, Pierre Rouanet, Christophe Halgand, Jenny Benois-Pineau, Florent Paclet, Daniel Cattaert, Pierre-Yves Oudeyer, Aymar de Rugy

**Affiliations:** ^1^Institut de Neurosciences Cognitives et Intégratives d'Aquitaine, UMR 5287 CNRS & Univ. Bordeaux, Bordeaux, France; ^2^Pollen Robotics, Bordeaux, France; ^3^Laboratoire Bordelais de Recherche en Informatique, UMR 5800, CNRS & Univ. Bordeaux & Bordeaux INP, Talence, France; ^4^Inria Bordeaux Sud-Ouest, Talence, France; ^5^Centre for Sensorimotor Performance, School of Human Movement and Nutrition Sciences, University of Queensland, Brisbane, QLD, Australia

**Keywords:** robotic arm, humanoid robot, research testbed, 3D printing, open-source, rehabilitation engineering

## Abstract

To this day, despite the increasing motor capability of robotic devices, elaborating efficient control strategies is still a key challenge in the field of humanoid robotic arms. In particular, providing a human “pilot” with efficient ways to drive such a robotic arm requires thorough testing prior to integration into a finished system. Additionally, when it is needed to preserve anatomical consistency between pilot and robot, such testing requires to employ devices showing human-like features. To fulfill this need for a biomimetic test platform, we present Reachy, a human-like life-scale robotic arm with seven joints from shoulder to wrist. Although Reachy does not include a poly-articulated hand and is therefore more suitable for studying reaching than manipulation, a robotic hand prototype from available third-party projects could be integrated to it. Its 3D-printed structure and off-the-shelf actuators make it inexpensive relatively to the price of an industrial-grade robot. Using an open-source architecture, its design makes it broadly connectable and customizable, so it can be integrated into many applications. To illustrate how Reachy can connect to external devices, this paper presents several proofs of concept where it is operated with various control strategies, such as tele-operation or gaze-driven control. In this way, Reachy can help researchers to explore, develop and test innovative control strategies and interfaces on a human-like robot.

## 1. Introduction

While robotic systems keep improving in terms of motor capabilities thanks to progress in mechatronics, developing control strategies and interfaces allowing a human to harness the full potential of an advanced robotic arm proves to be a key challenge in the field of humanoid robotics and in particular, rehabilitation engineering. Indeed, user surveys and reviews (Biddiss and Chau, 2007; Cordella et al., 2016) have already revealed that the lack of functionality and the necessity of a long and difficult training were some main reasons behind upper-limb prosthesis abandonment. As examples drawn from some of the most advanced devices currently on the prosthesis market, Michelangelo (Ottobock) and i-limb quantum (Touch Bionics) hands include too many actuators for an amputee to operate them independently, and their control relies a lot on pre-programmed grip patterns. Even in the case of an able-bodied human, the gap between robotic devices' complexity and available command signals highlights the need for efficient and usable control interfaces and strategies.

To bridge this gap, researchers have investigated techniques to retrieve additional input data from the human. One of these solutions is the sensor fusion approach, which intends to combine measurements from multiple sensors running at once. This approach can be used with various devices and sensing modalities (Novak and Riener, 2015), whether vision-based, kinematic, or physiological. In particular, as object recognition from egocentric videos can help grasping actions for neuroprostheses (de San Roman et al., 2017), recent works explored how a robotic system could be controlled by fusing eye-tracking with EMG (Corbett et al., 2013, 2014; Markovic et al., 2015; Gigli et al., 2017) or ElectroEncephaloGraphy (EEG) signals (McMullen et al., 2014; Wang et al., 2015). Other works also investigated how Augmented Reality (AR) can be employed to provide relevant visual feedback about a robotic arm's state (Markovic et al., 2014, 2017), with the aim of improving the control loop.

Another approach to overcome this limit is to reduce the need for command signals, by making the robotic system take charge of part of its own complexity. In this way, techniques are developed to allow a human to drive a robot through higher-level, task-relevant commands instead of operating the robot directly in actuator space. A common implementation of this approach is to perform endpoint control through Inverse Kinematics (IK), which convert command signals from the 3D operational space into the actuator space. IK solving is a key research topic in the whole field of robotics, including autonomous humanoid robotics (Bae et al., 2015; Rakita et al., 2018), but can also be employed to manage the kinematic redundancy of human-driven robots (Zucker et al., 2015; Rakita et al., 2017; Meeker et al., 2018).

To evaluate the performance of control techniques, virtual reality (VR) has been employed for more than a decade (Hauschild et al., 2007; Kaliki et al., 2013; Phelan et al., 2015; Blana et al., 2016). Recently, this approach also benefits from the increasing availability of VR development kits on the market, e.g., Oculus (Facebook Technologies, LLC) and VIVE (HTC Corporation), making it easier for researchers to integrate a virtual test environment into their experimental setup. However, a robotic system simulated within a virtual environment would not behave the same way as a physical device, inherently subject to mechanical limits and imperfections. Indeed, VR setups usually implement a simplified device (e.g., ideal, friction-less actuators) in a simplified context (e.g., ignoring gravity). As a result, conclusions drawn from assessments performed in a virtual test environment may not be directly applicable to an actual robot.

On the other hand, some researchers use actual robotic arms to get more realistic data from the testing phase. Works from the literature are found to employ both commercially available devices (Rakita et al., 2017; Meeker et al., 2018) and prototype systems (McMullen et al., 2014; Bae et al., 2015) in their research. More specifically, in the field of prosthetics, many multi-DoF devices have been developed as experimental prototypes, such as the UNB hand (Losier et al., 2011), the Yale hand (Belter and Dollar, 2013), and the SmartHand (Cipriani et al., 2011).

Among such research devices, the ones developed by Dawson et al. (2014) and Krausz et al. (2016) were designed with the aim of being inexpensive and easily available to other researchers, as open-source systems including 3D-printed parts. Indeed, as 3D-printing allows to produce complex and custom shapes in small batches at a low cost, this manufacturing technique is useful when developing products at the prototype stage. Besides, the fact that the same parts can be produced by many different 3D printers participates notably in the shareablity of these designs.

In this paper, we present Reachy, a life-size test platform to be used by researchers to explore, develop, and test control strategies and interfaces for human-driven robotics. Relying on technical solutions drawn from similar works, we aimed at designing a robot that would be **affordable**, **shareable**, and “**hackable**” compared to high-end prototypes or commercially available robotic arms; but also more **human-like** than industrial-grade robots. Indeed, Reachy benefits from its closeness to a human arm in terms of scale and shape, as well as motor features and joint structure. Additionally, even though its use cases are not limited to this field, this robotic platform is primarily intended for applications in prosthetics and rehabilitation engineering.

## 2. Robot Design

### 2.1. Design Principles

Reachy was created with the aim of providing researchers with a robotic platform on which to test control interfaces and strategies that would be employed to drive a robotic arm. In order to make the robot a relevant tool in the field of rehabilitation technologies, its structure puts the emphasis on human-likeness. Indeed, Reachy is meant to emulate the behavior of a life-size human upper limb, while being fixed at shoulder level on an unmovable support.

Besides, another major requirement of Reachy's design was to ensure that the robot is suitable for a variety of applications ranging from neuroprostheses to teleoperated manipulators. Thus, in order for Reachy to be a versatile platform, we intended to make it extensively customizable, as well as easily and broadly connectable. Ensuring extensive experimental reproducibility in this context requires the platform to allow for thorough hardware modifications, as well as the sharing of said modifications within the scientific community. Therefore, we chose to develop Reachy's design on the following principles and technical solutions: **3D-printed** plastic skeleton parts; **off-the-shelf actuators**, mechanical components and electronics; **free and open-source sharing** of both software and hardware resources.

Reachy was designed by the creators of the Poppy project (Lapeyre et al., 2014), a family of robots for research, art and education that rely on a common software and hardware architecture, but display a variety of shapes, features and purposes. In particular, the first robot of this family, Poppy Humanoid, was originally designed to investigate the role of morphology in biped locomotion (Lapeyre et al., 2013), thus generating the need for a platform whose parts could easily be redesigned, produced then assembled. The aforementioned design principles directly stem from the philosophy and technical solutions that drove Poppy's development.

### 2.2. Hardware

**Figure 1:**
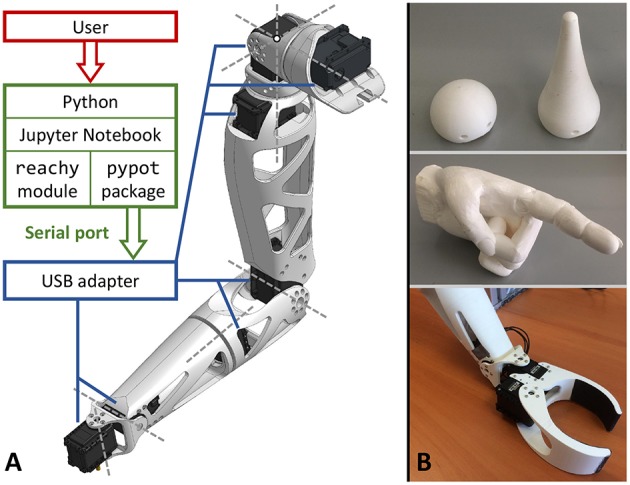
Reachy in its standard version. **(A)** Architectural diagram describing the software stack, from the high-level software interface to the motors. Dashed lines indicate the joints' rotation axes. **(B)** Currently available end-effectors. From top to bottom: spherical, conical, hand-shaped, and articulated clamp.

Reachy was initially developed as a “full-length” arm, that is to say, a prototype comprising the three segments of the human upper-limb, from shoulder to hand. In this “standard” version, Reachy weighs 1.4 kg and measures 60 cm from shoulder to wrist, with dimensions and proportions similar to those of a human adult's right arm. These prototypes have been equipped and tested with various end-effectors (see Figure 1B): a basic sphere, a jointless anthropomorphic hand, or a two-prong clamp providing a minimal grasping feature. Furthermore, as the robot is meant to be customized and “hacked”, Reachy users can adapt its distal end to fit an existing robotic hand chosen among available research prototypes (Losier et al., 2011; Belter and Dollar, 2013; Krausz et al., 2016). For instance, a new prototype featuring the Brunel hand (OpenBionics) as the end-effector has undergone development in order to expand the robot's features and capabilities.

In its standard version, the robot comprises seven independent DoF, each of them actuated by a dedicated motor. The first three motors operate the gleno-scapulo-humeral joint in a simplified way, by performing three consecutive rotations: shoulder flexion-extension, shoulder abduction-adduction, and humeral lateral-medial rotation. The three motors' rotation axes intersect at a single point, located at the center of the second motor's shaft (see Figure 1A). The shoulder assembly also comprises two roller bearings to facilitate the operation of the first and third DoF. In order for the robot to remain reasonably easy to model and build, this assembly does not reproduce the system of interdependent rotations and translations forming the gleno-scapulo-humeral complex, but still allows for a wide range of motion. The fourth and fifth motors operate respectively elbow flexion-extension and forearm pronation-supination, the latter being mounted with a ball bearing. Finally, the last two motors operate the wrist joint by performing consecutively radial-ulnar deviation and flexion-extension. Their respective rotation axes are orthogonal, however they do not intersect; instead, the two motors are linked by a short piece joining the forearm and end-effector. This interval between rotation axes shares some resemblance to that which separates a human's wrist joint axes, even though it is slightly too large because of the actuator size.

We chose to employ Robotis Dynamixel servomotors^1^ to actuate Reachy's seven DoF. These motors are all-in-one modules that provide a good trade-off between accuracy, speed and robustness in mechanical terms, as well as embedded sensors monitoring angular speed and position. They also allow the individual tuning of an internal Proportional-Integral-Derivative (PID) controller, maximum torque and mechanical compliance. Due to these features, Dynamixel servomotors enable a high level of modularity while being able to produce rich motor behaviors. For that matter, this range of actuators is commonly used in the field of robotics, including humanoid robots (Ha et al., 2011; Ly et al., 2011; Hild et al., 2012; Schwarz et al., 2013; Dawson et al., 2014). Apart from actuators, only few additional mechanical components are needed to assemble Reachy's joints, namely: the three aforementioned bearings, and transmission wheels to insert on each servomotor's shaft. In particular, as all these actuators include an individual gearbox, Reachy's assembly does not require extra reduction mechanisms for joint actuation.

Three different models of Dynamixel motors are included in Reachy's standard version. The most powerful one is an MX-106 and operates the most proximal DoF of the robot, shoulder flexion-extension, while the shoulder's remaining DoF and elbow joint are actuated by MX-64. As these four joints support the heaviest loads while the robot is put in motion, the corresponding motors were chosen accordingly. The forearm and wrist joints, which do not require as much power, are operated by AX-18, lighter and smaller than MX servomotors, so that the robot's weight distribution leans toward the proximal end.

Regarding the robot's skeleton, the limbs' design relies on a trellis-like structure to reduce the weight and keep assembly simple, by providing easy access to screw holes. This open, low-density structure also improves motor heat dissipation thanks to freer air circulation. Prototypes and current versions of Reachy were printed in polyamide or Poly-Lactic Acid (PLA), two materials commonly used in the additive manufacturing industry. Their low cost, availability and compatibility with most desktop 3D printers make them ideal for prototyping, while their durability and printing resolution make them adequate for finished products with good quality standards.

### 2.3. Electronics and Software

Reachy's motors are connected with each other in a series using three-pin connectors and powered by a pair of 12 V × 5 A power supply units, for a total power of 120 W. At one end of the series, a USB adapter allows for plugging into a computer. The robot is then controlled through a serial port with a software interface called Pypot, which handles the communication with Dynamixel servomotors to drive the robot, e.g., sending motor commands, retrieving data from embedded sensors. This architecture is illustrated in Figure 1A.

Developed as part of the Poppy project, this software base is common to the whole Poppy-Reachy family of Dynamixel-powered robots. Following an open-source approach, Pypot was entirely written in Python in order to enable cross-platform deployment, as this language is compatible with most desktop operating systems as well as some embedded systems for single-board computers. Python programming also allows for fast development by emphasizing code readability and conciseness, so that developers can efficiently produce clear programs whether their project is of small or large scale. Besides, Reachy users can take advantage of numerous Python libraries dedicated to scientific computing, and already in use within the scientific community. This allows them to combine Reachy's features with techniques such as signal processing or machine learning, without having to resort to other languages or software.

While its open-source nature provides expert programmers with extensive freedom over the system, Pypot is also intended to be accessible to beginners. In particular, it provides high-level motor commands over the joints' angular positions and mechanical compliance, so that any user can program a trajectory and put the robot in motion with only a few lines of code (see Supplementary Material). Additionally, tutorials are provided to Reachy users in the form of Jupyter notebooks (Kluyver et al., 2016), which are interactive development supports combining source code, formatted text, plots, and graphical input/output widgets. Jupyter notebooks can be created from a Web navigator and don't require any dedicated code editor. As a result, this software environment is accessible enough to allow Poppy robots to be currently used as educational platforms in several middle and high schools^2^.

**Figure 2:**
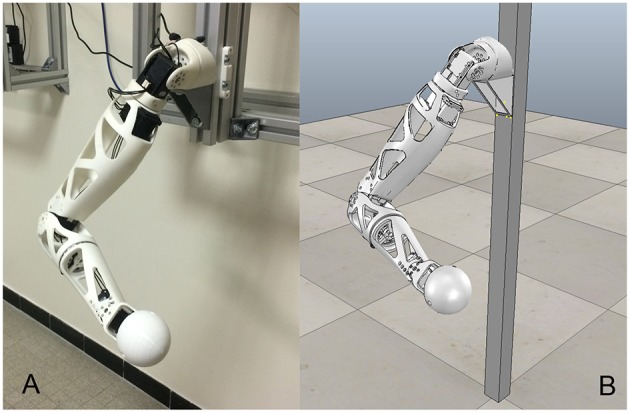
An actual Reachy robot **(A)** and its simulated counterpart **(B)**, set in the same posture.

Pypot also includes features to operate a virtual robot within the simulator V-REP (Freese, 2015), as illustrated in Figure 2. In this way, users can experiment and verify their developments on a simulated Reachy before deploying them on an actual robot in a physical setup. Migrating from a simulated to an actual robot, and vice versa, does not require any modification on the source code apart from a single keyword while configuring the connection to a robot.

### 2.4. Features

#### 2.4.1. Motor Performance

Reachy's motors can sustain up to 10 min of continuous operation and are able to work for as long as a full day when tasked with short, out-of-charge movements alternating with short resting periods. They provide a payload capacity of about 500 g at endpoint level, that the robot can handle for a few minutes. Their embedded load and temperature sensors also allow to automatically trigger resting phases, in order to prevent overheating during prolonged operation or after exposing the robot to significant strain. As a result, Reachy can be programmed to work autonomously for extended periods of time without putting the actuators at risk.

Out of charge, Dynamixel motors can reach a maximum speed of 500°/s and a maximum acceleration of 10,000°/s^2^. When they operate in their nominal angular speed range, their performance allows the robot's joint to reach their goal positions with a delay from 50 to 100 ms. This responsiveness makes it thus possible to develop real-time control schemes within which a human is continuously driving the robot. As a consequence, the robot can move its endpoint safely at a speed up to 2 m/s, with an acceleration of 10 m/s^2^.

Thanks to its three DoF at shoulder level, Reachy's full-length version can perform complex movements and postures in a wide range of action in the 3D space. As a result, Reachy benefits from having a workspace similar to that of a human adult's arm, within a 65 cm-radius sphere centered on its shoulder.

#### 2.4.2. Application in Prosthetics

Thanks to its human-like shape and joints, Reachy is suitable for applications in the field of upper-limb rehabilitation engineering, as a life-size test platform. Indeed, Reachy can emulate the behavior of a prosthetic arm in order to test and validate control schemes before implementing them on a genuine prosthesis. In this context, it also benefits from being notably cheaper than most commercially available upper-limb prostheses, thanks to its hardware architecture.

Indeed, 3D-printing technology has already been employed to create numerous arm prosthesis prototypes, whose designs are being developed by creators ranging from DIY enthusiasts and hobbyists, to researchers and engineers, as detailed in ten Kate et al. (2017). The fact that more than half of these 3D-printed devices' designs are shared online and available for free, shows how these creators take advantage of the interoperability of most desktop printers. This review also highlights how the production cost of these devices is one of the decisive aspects that sparkled the growth of this category of prosthetic devices, so much so that some 3D-printed prosthetic arms have recently went beyond the prototype stage and entered the market, such as the Hero Arm (OpenBionics).

Compared to the devices listed in this review, Reachy stands as one of the few models to address amputation above the elbow. Additionally, even though the predominance of transradial amputations among upper-limb disabilities explains the rarity of this type of prosthesis, Reachy is intended to enable research at multiple amputation levels. Indeed, the robot can be employed as a mockup device for any level of upper-limb amputation, when training a patient to produce muscle activity before being fit with a myoelectric prosthesis. This allows a patient to begin training even before being able to wear a prosthesis, e.g., while the stump is still cicatrizing. Obviously, such a training cannot replace experience with an actual prosthesis, especially because of the differences in terms of point of view, embodiment and perception of weight and inertia. Nonetheless, it can take place in a patient's rehabilitation as a complementary or preliminary training, with the aim of getting familiar with myoelectric control as well as motors' responsiveness and accuracy.

In this context, the patient's lost motor functions are emulated with Reachy's corresponding joints while the robot's more proximal actuators are locked in a given posture. In this way, the patient can practice performing appropriate muscle contractions and receive relevant feedback from the robot moving accordingly, following a given prosthesis control scheme. In a more advanced setup, the patient's residual limb movements can even be tracked and reproduced on these motors, instead of being locked. Such a setup could turn out to be useful as well for testing control strategies using residual limb motion as input signal to drive the prosthesis (Kaliki et al., 2013; Merad et al., 2016).

Regarding the end-effector, in respect to grasping with an arm prosthesis, a fixed wrist often requires the patient to perform extra shoulder and elbow movements to compensate for the lack of distal mobility. Thus, enabling wrist motion proves to be quite useful for a patient (Kanitz et al., 2018), as it enables a more natural and comfortable use. In this way, Reachy's 2-DoF wrist makes it suitable to address this aspect of prosthesis control. In combination with forearm rotation, these motor functions at wrist level allow the robot to put its endpoint in a wide variety of 3D orientations, enabling different grasping types depending on the item of interest.

**Figure 3:**
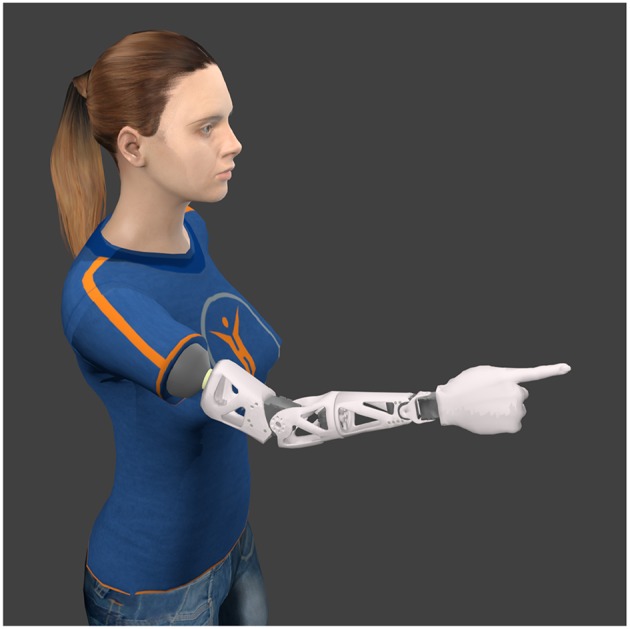
Virtual illustration of a possible evolution of Reachy: socket-mounted version worn by a transhumeral amputee.

Finally, Reachy's customizable architecture allows users to design, print and assemble custom fixings, so that a part of the robot can be mounted on an actual prosthetis socket or harness, and worn by an amputee (see Figure 3). Whether at transradial or transhumeral level, the robot's skeleton parts can be modified so that its dimensions are adjusted to the wearer's morphology, to fit best with the stump's anatomy as well as the sound limb's proportions. Obviously, Reachy is not meant to replace a prosthesis for daily use, but a socket-mounted version of Reachy could as well be employed for training patients with myoelectric control.

### 2.5. Comparison With Existing Robotic Arms

In order to put Reachy's performance and abilities in perspective with existing robotic devices, we compared it with several related robotic arms, considering various aspects and features.

The Bento Arm (Dawson et al., 2014) is a robotic arm employed in research and upper-limb rehabilitation to emulate a myoelectric prosthesis. It includes five joints from humerus to end-effector, each actuated by a Dynamixel motor. Its mechanical structure follows human-like shapes and proportions, and relies mostly on 3D-printed plastic parts. In this sense, this robot is very similar to Reachy, although the upper arm includes only a single DoF at humeral level, and none at shoulder level. As a consequence, its workspace is limited to a 22 cm-wide circular area centered on the elbow, for a payload capacity of only 300 g. This narrow range of motion and limited upper-arm actuation make the Bento Arm unsuitable for research on whole-arm movements, unless it is mounted on a prosthesis socket. As a rehabilitation device, it focuses on emulating a transradial prosthesis but appears to be inappropriate with respect to higher levels of amputation. In particular, it cannot be employed to study or reproduce coordinations between upper-arm joints.

The GummiArm (Stoelen et al., 2016) is an experimental bio-inspired robotic arm comprising 10 tendon-driven joints, actuated by 19 Dynamixel motors. With eight of its joints enabling variable stiffness, this robot can perform movements in a workspace similar to that of a human arm while being safe to physically interact with. Similarly to Reachy, its 3D-printed skeleton parts and open-source approach allow for replication and modification by users. However, its higher number of actuators and tendon-based mechanics make it more expensive (over 5,000$ in spare parts, over 11,500$ as a kit) as well as more suitable for research on bio-inspired actuation and compliant motor control than on rehabilitation engineering. Indeed, most upper-arm, elbow and forearm prostheses are actuated by a single motor per joint in a non-compliant fashion, instead of emulating human biomechanical structures.

The Modular Prosthetic Limb (MPL) (Johannes et al., 2011) is an experimental robotic upper-limb prosthesis, comprising 26 joints actuated by 17 motors. Its high-grade components and anthropomorphic design allow it to reach human-like strength in the wide range of motion offered by its joints. Compared to Reachy, this robot offers much better motor performance, such as a payload capacity of 15 kg and a joint speed of 120°/s. However, these abilities also come with a higher power consumption (24 vs. 5 V for Reachy) and a heavier weight (4.7 vs. 1.2 kg for Reachy).

The DLR Hand Arm System (Grebenstein et al., 2011) is an experimental bio-inspired robotic arm, now integrated to the humanoid robot David as its upper limb. It includes six DoF in the arm and 19 in the hand, actuated by a total of 52 motors. Its tendon-driven mechanical structure allows the robot to operate dexterously at a speed and in a workspace comparable to those of a human, making it clearly more capable than Reachy in terms of motor performance. However, its bidirectional antagonist joints require numerous motors and mechanical components, a dedicated transmission architecture and a dense electronics network managing both actuation and sensing.

Due to their price and complexity in terms of electronics and mechanics, these advanced robotic devices are much more difficult to replicate or customize in depth. In this sense, their users depend significantly on the robot's designers and makers to assemble, modify and repair it, whereas Reachy's design allows users to handle every step of the fabrication process. Its architecture is simple enough to allow non-experts to build it and connect it to a computer. Regarding control and interfacing, both of these robots rely on complex control architectures (Bridges et al., 2011; Grebenstein et al., 2011) running in Simulink, proprietary software owned by MathWorks, Inc. In this regard, the pieces of software operating these devices are less open and more difficult for a user to modify or adapt to a given use case. Conversely, Reachy benefits from its open-source software architecture providing many interfacing options, with a variety of command signals and external software tools.

Although Reachy does not compare to these advanced robots in terms of performance, its connectability and highly modifiable structure make it a suitable research platform. In this sense, we wish to promote Reachy as a complete platform combining both hardware and software characteristics fostering replication, customization and versatility. We are not aware of a similar robotic system that would offer as many possibilities, based on the comparison detailed in this section.

### 2.6. Sharing Philosophy

Reachy is developed in partnership with and distributed by Pollen Robotics^3^ as a fully open-source project. Besides, users willing to assemble the robot by themselves can buy all the hardware in spare parts, at a total price below 4,000$ for the standard version. Any laboratory can build their own Reachy, modify its components and customize it at will, on both hardware and software sides. This allows researchers to adapt the robot to their specific needs and interface it with their own devices and tools.

The source files from the Computer-Aided Design (CAD) models of the different printable parts are shared under the Creative Commons BY-SA license^4^ and available online^5^. The bill of materials and software components that are specific to Reachy are shared under the Lesser GNU General Public License^6^ and available online in the project's repository^7^. The Pypot library is shared under the GNU General Public License^8^ and available online in a dedicated repository^9^.

As Reachy relies on the same software and hardware architecture as Poppy robots, it is worth noting that its users can benefit from the help and contributions shared by the Poppy project's community on its repository^10^. Indeed, this community hub gives access to many tips regarding the different aspects of the robot, from configuring and assembling the servomotors to setting up the software tools and troubleshooting.

## 3. Proofs of Concept

In order to illustrate Reachy's interfacing capabilities, we developed several proofs of concept where the robot's features are combined or expanded with various external devices and software tools. All the proofs of concept described below were developed in Python, to further demonstrate the interfacing potential provided by this language.

### 3.1. Inverse Kinematics for Endpoint Position Control

Determining a set of motor angles that put a robot's endpoint at a target position in its operational space is a common problem in the field of robotic arms, and is usually referred to as the *Inverse Kinematics* (IK) problem. As it comprises seven independent DoF, Reachy typically displays *kinematic redundancy*, implying that there is an infinite number of distinct solutions to this problem for each reachable target position. Thus, in order to drive the robot's endpoint position to a given target, one needs to determine which set of angles to apply, among the infinity of possible sets. However, the numerical expression of this under-constrained geometrical problem is non-linear, which makes analytic solving impractical and costly in terms of computation.

#### 3.1.1. Local Optimization

Instead, a widespread method used by roboticists to solve IK problems is to employ local optimization. This method relies on a cost function, attributing a scalar value to any set of angles to quantify to what extent it is a good solution with respect to the IK problem: a lower cost means a better solution. Usually, this cost function is based on the distance between the target and the endpoint position, which can be analytically determined with the geometrical model of the robot. Then, through a step-by-step process, the optimization finds and returns a local minimum of this cost function, that should correspond to one of the sets of angles putting the endpoint at the required position.

We used the Python library IKPy (Manceron, 2015), a generic IK solver, to apply this method on Reachy. The robot's software resources include a Universal Robot Description Format (URDF) file describing Reachy's mechanical properties, such as the relative position and orientation of each joint and skeleton part. These geometrical data can then be imported with IKPy to build the corresponding kinematic chain, by going through the sequence of joints from the robot's base to its endpoint. In this way, this interfacing between IKPy and Reachy's software interface can be performed straight out of the box, and works as a standalone, without requiring any external device or specific hardware. As a result, combining IKPy's features with the motors' command options provides a new and easy way to control Reachy by sending 3D coordinates as commands, instead of joint angles. A code sample showing how to use IKPy with Reachy is available online^11^.

IKPy allows to set parameters for the optimization process (e.g., maximum iteration number, convergence tolerance) when calling it from another program. Thanks to these options available in the code, users can fine-tune the process in respect to the intended use case and available computing power. As an example, after fine-tuning our setup through a trial-and-error process, the model was able to reach a sub-centimetric accuracy with a computing time below 100 ms on a desktop computer. However, the kinematic chain employed with this method is a **theoretical** model of the robot and does not take into account the robot's weight and joints' mechanical play. On a physical robot, as actual motors are unable to reach the *exact* angular positions determined by IKPy, the endpoint tends to undershoot when driven with this method. To assess endpoint accuracy, the distance between the endpoint's actual position and its target was measured for eighty postures distributed in the robot's range; each measurement was performed after the robot moves for 1.5 s then is asked to hold the posture for 3.5 s. We obtained a mean distance to target of 87 mm (SD 23 mm), and also observed that the endpoint usually reaches positions located under the target. Indeed, position errors along the two cartesian horizontal axes are roughly centered on zero (mean <5 mm) whereas along the vertical axis, this error is subject to a notable offset (mean = 84 mm).

Nevertheless, this flaw is not blatantly noticeable if no visible object materializes the target position in the operational space. Besides, the vertical offset proves to be fairly consistent over time and reachable space. Therefore, in the context of a continuous endpoint position control, it can be dealt with during a calibration phase performed prior to the control phase. In this way, this interfacing between IKPy and Reachy can be conveniently employed in applications where there is no strong need for endpoint accuracy in the operational space.

To assess repeatability, the robot was tasked to perform several times the same movement while the endpoint's position was recorded with a motion tracking system (Optitrack V120 Trio, Natural Point Inc.). Firstly, the robot was tasked to travel accross a 40 cm-wide circle in a frontal plane, in 3.5 s. A comparison of the recorded trajectories showed that on keyframes distributed along the movement, for a given set of motor goals, the robot's resulting endpoint positions were spread within a 12 mm-radius sphere. Then, the robot was tasked to reach a given posture and hold it for several seconds before its endpoint position was recorded. Over ten repetitions of this movement, the positions were spread within a 5 mm-radius sphere. These results illustrate Reachy's ability to reach the same point in space in response to the same motor commands, in both static and dynamic contexts.

#### 3.1.2. Supervised Learning With an Artificial Neural Network

On another hand, this gap between a theoretical model and Reachy's actual functioning can be reduced by employing modeling techniques that do not intend to simulate the robot's *ideal* behavior. One of them consists in recording actual movements performed by the robot and using them as “ground truth” examples on which to perform supervised learning. The goal is to build a set of movements where both motor angles and endpoint coordinates are synchronously recorded, so that a supervised learning algorithm can emulate the actual relationship between these two quantities.

To apply this technique with Reachy, we first defined a set of robot postures through physical demonstration: with its motors set as compliant, the robot was manually placed in various configurations while embedded sensors recorded the joints' angles. Then, based on the recorded angles, the robot performed movements going from one of such demonstrated postures to another, while the Optitrack V120 recorded the actual endpoint position. We used an Artificial Neural Network (ANN) to perform supervised learning on the captured joint and endpoint data. ANNs are computational tools relying on elementary logical units called “neurons” and connected between them by weighted links, generally following a specific network architecture (Reed and Marks, 1998). For several decades, these tools have been used to perform supervised learning by tuning the weights of these links based on the training data. In the field of robotics, ANNs are typically employed to perform environment sensing or effector control, including IK solving (Bouganis and Shanahan, 2010; Duka, 2014; Almusawi et al., 2016).

Our results were obtained with a feed-forward multi-layer perceptron including two fully connected hidden layers of, respectively 64 and 128 neurons. We employed the TensorFlow (Abadi et al., 2015) backend and Keras (Chollet, 2015), a Python programming interface for ANNs to implement and train this network to perform IK solving, that is to say: take endpoint coordinates as input and return corresponding joint angles as output.

Relatively to using IKPy, implementing this method is more demanding and requires to carry out the previously described two-phase data acquisition process using motion capture equipment. However, as this technique is based on movements performed by the physical robot instead of a mechanically perfect model, the ANN implicitly takes into account the deviations between the motor commands sent to the robot and the angles actually reached by the motors. As a result, this method proved to be more accurate than the local optimization method with an actual robot (mean distance to target = 25 mm, SD 11 mm). In particular, it does not suffer from the aforementioned vertical offset, as the position error along the vertical axis isn't more off-centered than along horizontal axes (mean < 14 mm for all three axes). On another hand, the computing time required to perform a single IK solving with this method remained consistently under 1ms, proving it to be much faster than local optimization.

Besides, building the training set through manual demonstration of postures allows users to deliberately introduce a bias in favor of a certain type of posture. In this way, such a bias would be implicitly learned and emulated by the network, as its output would be, by design, similar to the training set's postures. For instance, if one only records postures with a horizontal hand and palm facing downwards, virtually all joint angles returned by the network should correspond to postures displaying that same characteristic.

Regarding network structure, we noticed that adding more hidden layers or increasing their size does not draw significant benefits and can even result in the network overfitting the examples, while notably increasing the time required to train it. Based on these observations, we hypothesize that more complex network architectures, such as convolutional or recurrent networks, may not be appropriate for the solving of this IK problem.

As a conclusion on the topic of IK solving for Reachy, we presented here two methods with notable differences regarding accuracy, practicality or convenience. These methods also illustrate how Reachy benefits from being connectable and customizable, in the way that various solutions can be employed to provide similar features, so that users can choose a solution suitable for their needs. Other approaches could be employed to perform endpoint position control, either based on existing techniques from the literature, or developed *ad hoc* with more specific requirements.

### 3.2. Tele-operation

Based on the endpoint position control feature made available by these IK solving techniques, we developed a second proof of concept, which we refer to as “tele-operation.” The goal of this proof of concept is to provide users with an intuitive and transparent way to drive the robot in real time, that would not require them to send explicit, quantitative commands such as joint angles or endpoint coordinates. With this aim, the tele-operation driving mode works by continuously tracking a subject's hand trajectory and simultaneously mapping it on the robot's hand, considered its endpoint.

**Figure 4:**
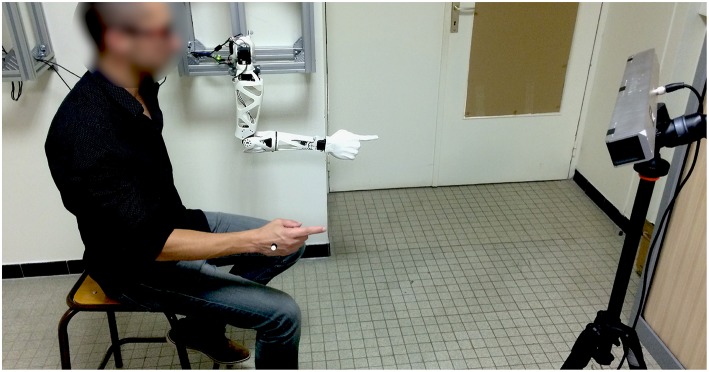
Tele-operation setup, shown during the calibration phase. Subject and robot are placed in the same posture while the Optitrack system (on the left) records the coordinates of the reflecting marker placed on the subject's hand. See this driving mode in operation at https://www.youtube.com/watch?v=Oa9mHMoDtYI.

Our implementation of this driving mode makes use of the Optitrack V120 Trio as the motion tracking system, to determine the 3D position of a marker placed on the hand. We interfaced the device with a Python program using OptiRX (Astanin, 2016) to retrieve marker data in real time at 120 Hz. Before the control phase, a calibration is performed to set the relation between the subject's reference frame, in which marker data is expressed, and the robot's reference frame, in which endpoint target coordinates must be expressed. Then, both the robot and subject's arms are placed in the same initial posture: humerus along the body and elbow flexed at 90° (see Figure 4). In this posture, the subject's and robot's hand positions are saved in order to work as origin points in their respective frames. At each instant of a 10 Hz control loop, the former is used to compute the instantaneous displacement vector of the subject's hand, then the latter is used to compute the robot's hand target position, by mapping this vector in the robot's frame. Finally, using an IK solving method, Reachy is put in motion so that its endpoint goes toward this instantaneous target.

As a result, the subject can drive the robot by performing natural arm movements, observing how Reachy mirrors them and using this visual feedback to adjust the robot's motion. Obviously, the processing time as well as the fact that the motors cannot instantly reach the goal angles sent as commands, introduce a lag between its endpoint's movement and the subject's hand trajectory. In the current setup, this lag is usually comprised between 350 and 450 ms. This proof of concept illustrates how one can implement a control strategy with Reachy, that is: a way to put it in motion based on data acquired by external devices. It also demonstrates how Reachy can be controlled in a real-time fashion, while performing smooth and steady movements.

A video clip showing a subject driving the robot in tele-operation mode is available online^12^.

### 3.3. Gaze-Driven Control

Following on from vision-based assistive devices, we developed a second proof of concept to explore how eye movements and gaze behavior could be employed as a source of commands to put Reachy in motion. With this aim, we tried to interface the robot with eye-tracking and image processing tools, in order to allow a subject to drive Reachy by moving only their eyes instead of their limbs. Eye tracking is a category of techniques aiming at measuring eye movements or gaze direction, whether for observation purposes or as input in an interactive setup (Duchowski, 2003). In the field of robotics, eye-tracking techniques have recently been employed to control robotic arms, especially with applications in rehabilitation and assistive technologies (Frisoli et al., 2012; McMullen et al., 2014; Hortal et al., 2015).

**Figure 5:**
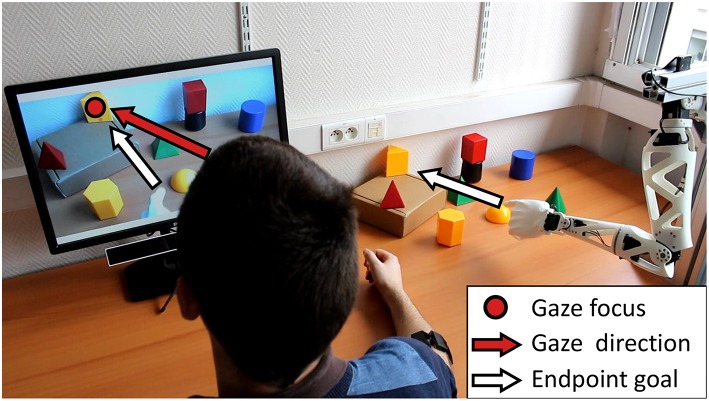
Gaze-driven control setup. On the right, various objects are located in front of a left-handed version of Reachy, and the resulting scene is filmed by the camera placed over the robot's shoulder. Its video feed is shown on the screen on the left, under which the eye-tracker is placed. The subject wears the Myo armband on the right forearm to trigger a movement by the robot. See this driving mode in operation at https://www.youtube.com/watch?v=qloR67AaqQ4.

The resulting setup relies on a camera filming a scene in front of the robot, and a computer screen displaying its video stream to a subject. The camera is placed so that the scene matches with the robot's reachable space, and hand-sized objects of various colors and shapes are located within its range. They are placed so that no visual occlusion occurs from the point of view of the camera, and no physical obstruction occurs when the robot moves its endpoint toward an object. In this setup, the screen acts as a 2-dimensional proxy between the robot's operational space and the subject's field of view, in order to use eye-tracking technology in a simpler context than 3D space. We employed the GP3 HD eye tracker (Gazepoint) to locate the focus of the subject's gaze on the plane of the screen, and identify the corresponding object in the robot's reaching space (see Figure 5). Then, the robot can be put in motion toward this object's position, either using pre-recorded postures, or a combination of inverse kinematics and computer vision in the scene in front of the robot.

Finally, using a Myo armband (Thalmic Labs Inc.), we also integrated a basic form of myoelectric control to this setup. This measuring device allows for the detection of a specific muscle activation pattern, that can be interpreted as a command signal. In this way, the subject can perform, for instance, a voluntary co-contraction of forearm muscles to trigger a movement by the robot. Whenever such a signal is detected, the object on which the subject's gaze is focused is identified by the eye-tracking system, and Reachy is put in motion accordingly.

It is worth noting that the processing of gaze data performed to identify the object of interest remains very basic in this simplified setup. In a daily life context, the subject's posture would be unconstrained. Furthermore, the environment the subject acts in, performing its instrumental activities of daily living, is cluttered. The distractors and scene changes provoke saccades. When maintaining gaze on the target object, the geometry in a dynamic scene is also unstable due to micro-saccades. This is why a filtering of gaze fixation signal along the time is needed (González-Díaz et al., 2019). Moreover, today a localization of objects in a gaze-predicted area can be solved together with an object-recognition task, employing powerful deep CNN classifiers. This allows for more precise object localization and also adaptation to the scene dynamics due to the unconstrained motion of the subject.

The source code employed in this proof of concept is available online^13^. A video clip showing a subject performing gaze-driven control is available online^14^.

## 4. Conclusion and Perspectives

Reachy, a seven-DoF human-like robotic arm, was developed to act as a test platform for research on human-driven robotic arms. Following an open-source approach, its design was elaborated to allow for easy sharing and low fabrication cost, with the purpose of enabling extensive customization in a wide variety of applications. Software and hardware resources were made available online so that researchers and laypeople can build a Reachy robot and integrate it in their own experiments and projects.

In the short term, immediate applications of Reachy include the exploration, development and testing of control strategies and interfaces for robotic arms. In this way, several prototypes were produced and proofs of concepts were developed in order to illustrate potential use cases in various fields in relation with human-driven robotics. As a broadly connectable platform, it allows to investigate hybrid control strategies, combining biomechanical signals with motion- or eye-tracking tools and computer vision techniques (de San Roman et al., 2017; González-Díaz et al., 2019). Reachy can also help study how vision-based control strategies would help driving rehabilitation devices, such as an assistive arm fixed to a wheelchair, for use by patients suffering from Spinal Cord Injury (SCI) (Corbett et al., 2013, 2014).

On the longer term, Reachy can be employed as a mockup device for research and training with upper-limb neuroprostheses. In particular, it can help patients get familiar with muscle signal production and myoelectric control prior to being fit with an actual arm prosthesis. Additionally, thanks to its motors' control options, Reachy is suitable to address different levels of amputation, by employing separate control modes to drive proximal and distal joints. For instance, as a way to emulate transhumeral amputation, Reachy can be controlled through a “hybrid” teleoperation mode where shoulder joints reproduce a patient's actual shoulder motion while the other motors are driven with a separate, artificial control strategy. Similar approaches were investigated in recent works (Kaliki et al., 2013; Merad et al., 2016), where natural shoulder motion (performed by a subject) is used to infer artificial elbow and/or wrist motion (performed by a virtual avatar or a wearable prosthesis). As a general conclusion, Reachy can prove to be a versatile device suitable for applications with multiple approaches for the control of an upper-limb neuroprosthesis.

## Data Availability

All datasets generated for this study are included in the manuscript and/or the Supplementary Files.

## Author Contributions

SM participated in the development of proofs of concept under the supervision of P-YO and AR, and wrote the paper. ML and PR designed the robot and participated in the development of proofs of concept. CH participated in the development of proofs of concept. JB-P, FP, and DC provided inputs for paper writing. All authors approved the final version.

### Conflict of Interest Statement

ML and PR are affiliated to Pollen Robotics, a company with financial interests in the system presented in this work, as its primary supplier. The remaining authors declares that the research was conducted in the absence of any commercial or financial relationships that could be construed as a potential conflict of interest.

## Footnotes

^1^http://en.robotis.com/subindex/dxl_en.php

^2^http://perseverons.espe-aquitaine.fr/sp6-robotique-inria/

^3^https://www.pollen-robotics.com/en/our-products/humanoid-robot-arm-reachy/

^4^http://creativecommons.org/licenses/by-sa/4.0/

^5^http://cad.onshape.com/documents/66388ae9c63cef53d76acd77/w/68c2411483d5bc65c7f54234/e/581d46ba9b8ee98de9d636ee

^6^http://gnu.org/licenses/lgpl-3.0.html

^7^http://github.com/pollen-robotics/reachy

^8^http://gnu.org/licenses/gpl.html

^9^http://github.com/poppy-project/pypot

^10^http://github.com/poppy-project

^11^http://github.com/pollen-robotics/reachy/blob/master/doc/notebook/Kinematics.ipynb

^12^https://www.youtube.com/watch?v=Oa9mHMoDtYI

^13^http://github.com/pollen-robotics/reachy/blob/master/applications/video_gaze.py

^14^https://www.youtube.com/watch?v=qloR67AaqQ4
