# Supplementary material for: Reachy, a 3D-Printed Human-Like Robotic Arm as a Testbed for Human-Robot Control Strategies
Source: Front Neurorobot. 2019 Aug 14;13:65. doi: 10.3389/fnbot.2019.00065 (PMC6703080; doi:10.3389/fnbot.2019.00065)
Supplement: Supplementary file 1 [file Data_Sheet_1.pdf]

frontiers

Supplementary Material

1 PYTHON CODE SAMPLE

This appendix presents a code sample demonstrating the compactness of basic commands and options to control Reachy.

```python
from reachy import Reachy
robot = Reachy()  # Connect to the robot
robot.compliant = False  # Stiffen its motors

# 90° degree elbow flexion in 2 seconds
robot.elbow_pitch.goto_position(-90, 2)

# Retrieve some data from embedded sensors
angle = robot.elbow_pitch.present_position
temp = robot.elbow_pitch.present_temperature

bounds = {}
for m in robot.motors:
    # Set max angular speed allowed
    m.moving_speed = 300
    # Store angular bounds by motor name
    bounds[m.name] = (m.lower_limit,
                      m.upper_limit)

# Adjust settings selectively on the three first motors (shoulder)
for m in robot.motors[:3]:
    m.pid = (3, 1, 1)     # PID gains
    m.torque_limit = 75  # Max load allowed
```

2 VIDEO CLIPS

2.1 Tele-operation

Link: https://www.youtube.com/watch?v=Oa9mHMoDtYI

Description: Demonstration of the tele-operation setup, using the V120 Trio motion tracker and IKPy

2.2 Vision-driven control

Link: https://www.youtube.com/watch?v=qloR67AaqQ4

Description: Demonstration of the vision-driven control setup, using the GP3 HD eye tracker, the Myo armband and a set of small objects
